# Regulation of leaf hydraulics: from molecular to whole plant levels

**DOI:** 10.3389/fpls.2013.00255

**Published:** 2013-07-15

**Authors:** Karine Prado, Christophe Maurel

**Affiliations:** Biochimie et Physiologie Moléculaire des Plantes, UMR 5004 CNRS/UMR 0386 INRA/Montpellier SupAgro/Université Montpellier 2Montpellier, France

**Keywords:** aquaporin, hydraulic conductance, leaf growth, veins, xylem

## Abstract

The water status of plant leaves is dependent on both stomatal regulation and water supply from the vasculature to inner tissues. The present review addresses the multiple physiological and mechanistic facets of the latter process. Inner leaf tissues contribute to at least a third of the whole resistance to water flow within the plant. Physiological studies indicated that leaf hydraulic conductance (*K*_leaf_) is highly dependent on the anatomy, development and age of the leaf and can vary rapidly in response to physiological or environmental factors such as leaf hydration, light, temperature, or nutrient supply. Differences in venation pattern provide a basis for variations in *K*_leaf_ during development and between species. On a short time (hour) scale, the hydraulic resistance of the vessels can be influenced by transpiration-induced cavitations, wall collapses, and changes in xylem sap composition. The extravascular compartment includes all living tissues (xylem parenchyma, bundle sheath, and mesophyll) that transport water from xylem vessels to substomatal chambers. Pharmacological inhibition and reverse genetics studies have shown that this compartment involves water channel proteins called aquaporins (AQPs) that facilitate water transport across cell membranes. In many plant species, AQPs are present in all leaf tissues with a preferential expression in the vascular bundles. The various mechanisms that allow adjustment of *K*_leaf_ to specific environmental conditions include transcriptional regulation of AQPs and changes in their abundance, trafficking, and intrinsic activity. Finally, the hydraulics of inner leaf tissues can have a strong impact on the dynamic responses of leaf water potential and stomata, and as a consequence on plant carbon economy and leaf expansion growth. The manipulation of these functions could help optimize the entire plant performance and its adaptation to extreme conditions over short and long time scales.

## INTRODUCTION

The growth of plants is critically dependent on two key physiological processes that occur in leaves: gas exchange through stomata and carbon fixation in the photosynthetic tissues. To operate optimally, these processes require a well-balanced hydration status of the leaf.

The water status of plant leaves is dependent on both stomatal regulation and water supply from the vasculature to inner tissues. The present review addresses the multiple physiological and mechanistic facets of the latter process. Following uptake by root and transport to shoots via vascular tissues, water (xylem sap) is delivered throughout the whole leaf lamina, before evaporating in the substomatal chambers and diffusing through the stomata (Sack and Holbrook, 2006). A small portion of the leaf water flow is used to support expansion growth (Pantin et al., 2012).

Water transport in leaves is therefore mediated through a complex network of hydraulic structures. The organization of this network is dictated by independent structural constraints, for optimizing sap delivery and other leaf functions such as light harvesting (Brodribb and Feild, 2010). Beyond anatomical features, our understanding of most of molecular and genetic mechanisms involved in leaf water transport is incomplete. In particular, the respective contributions of the vessels and the living tissues to water transport as well as the pathways used by water in the latter tissues are extensively studied. Recent advances about the mechanisms that allow the adjustment of leaf hydraulics in response to developmental and environmental factors will also be presented.

## LEAF HYDRAULIC CONDUCTANCE: A HIGHLY VARIABLE PARAMETER

### LEAF HYDRAULIC CONDUCTANCE

Following its delivery from the stem as xylem sap, liquid water flows through veins, and crosses the xylem parenchyma, bundle sheath, and mesophyll tissues before evaporating in leaf air spaces and substomatal chambers. Thus, water transport in leaves involves two states of water, liquid and gaseous.

The transport of liquid water in inner leaf tissues, which is the object of the present review, is governed by classical flow equations used in plant water relations (Steudle, 1989). These equations tell us that water transport intensity is linearly linked to both the driving force (water potential gradient) between the petiole and the substomatal cavity and the water transport capacity (hydraulic conductance) of the leaf (*K*_leaf_; Sack and Holbrook, 2006). *K*_leaf_ is therefore a key physiological parameter to address the transport of liquid water in the leaf, while excluding the contribution of stomata to water vapor diffusion. *K*_leaf_ integrates all water transport paths working in parallel or in series within the inner leaf tissues, each having its own physical characteristics.

### TECHNIQUES FOR MEASURING WATER TRANSPORT IN WHOLE LEAVES

Experimentally, *K*_leaf_ is determined as the ratio of water flow rate through the leaf to the driving force, that is, the water potential difference between the petiole and leaf lamina (ideally, the substomatal chambers). *K*_leaf_ is usually normalized by leaf area (Sack and Holbrook, 2006). At the whole leaf level, three major techniques have been developed to measure *K*_leaf_.

The evaporative flux method (EFM) is the most commonly used. It relies on the relationship that exists, under steady state conditions, between the flux of transpiration across the plant or an excised leaf and the corresponding drop in water potential (Martre et al., 2002; Sack et al., 2002). *K*_leaf_ is deduced from the ratio of transpiration flow to the difference of water potential between the stem and the leaf. In practice, water potentials are measured in a fully transpiring leaf and in a leaf covered with a bag to locally prevent any transpiration. The latter leaf reports on the stem water potential.

The high pressure method (HPM) requires a flow of solution to be pushed using a pump, from the petiole throughout the leaf (Sack et al., 2002; Tyree et al., 2005). Alternatively, an excised leaf or rosette can be inserted into a pressure chamber whereby a flow of solution is pressed through the stomata and exits the leaf through the hypocotyl section (Postaire et al., 2010). *K*_leaf_ can be deduced from the flow vs. pressure relationship. It has been argued that stomatal constrictions could dominate the measured *K*_leaf_. However, Poiseuille’s law indicates that, by contrast to vapor phase transport, the pore apertures must represent a negligible resistance under conditions of liquid flow. These assumptions were supported experimentally in walnut (*Juglans regia*) which leaves showed a marked stomatal closure in response to abscisic acid (ABA), without any alteration in *K*_leaf_ (Tyree et al., 2005) and in model species *Arabidopsis thaliana* which rosette hydraulic conductivity was increased under darkness, simultaneously to stomatal closure (Postaire et al., 2010).

The vacuum pump method (VPM) represents the third type of *K*_leaf_ measuring method. In this case, water enters an excised leaf through its sectioned petiole. The leaf blade is carefully maintained at saturating water vapor but subjected to vacuums of different intensities. Thus, water is pulled by suction, in the absence of any vapor pressure deficit and the measured *K*_leaf_ mostly reflects a liquid phase conductance of inner leaf tissues (Sack et al., 2002).

There are still ongoing discussions about the respective validity of these three types of *K*_leaf_ measurement methods (Rockwell et al., 2011). For instance, water potential measurements required for the EFM have many pitfalls. However, this method is performed in conditions whereby water evaporates in the leaf airspaces and diffuses from the stomata, and it has been argued that, with regard to other methods, EFM most closely reports on the natural pathway of water in leaves (Sack and Scoffoni, 2012). In contrast, the HPM and VPM may not yield *K*_leaf_ values that reflect the *in vivo* context, since a flow of water is driven through the leaf at higher hydrostatic pressure gradients than ambient. In addition, during HPM measurements (and perhaps to some degree with the VPM), the leaf or rosette is flooded with a liquid solution and leaf airspaces rapidly become infiltrated. This may create novel pathways for water movement, in addition to those utilized during transpiration. Yet, several comparative studies, including one with six woody angiosperm species, showed that similar *K*_leaf_ values (with differences around 10%) could be determined by the three methods (Sack et al., 2002). From this, it was inferred that the mesophyll pathway that may be shunted when using the HPM may be of negligible resistance (Sack et al., 2002).

### LEAF HYDRAULIC CONDUCTANCE VALUES ACROSS PLANT SPECIES

A comprehensive set of *K*_leaf_ data has now been collected in the whole plant kingdom. These studies revealed that *K*_leaf_ is highly variable, by up to 65-fold across plant species (Sack et al., 2005).

These studies also established that, with respect to roots and stems, leaf tissues can represent a substantial part of the inner resistance to whole plant water flow. Within a sample of 34 species, the leaf contributed on average a third of the whole plant resistance (Sack et al., 2003) but in some cases it could represent up to 98% of this resistance (Sack and Holbrook, 2006). Of outstanding interest for the physiologist is also the observation that *K*_leaf_ can be highly variable and dynamic during plant life. Thus, *K*_leaf_ depends on the anatomy and developmental stage of the leaf; it can also vary according to plant growing conditions, over a wide range of time scales, from minutes to months.

## VARIATION OF LEAF HYDRAULIC CONDUCTANCE IN RESPONSE TO DEVELOPMENTAL AND ENVIRONMENTAL FACTORS

### DEVELOPMENT

*K*_leaf_ shows dynamic changes over the whole leaf lifetime, with patterns specific for each species (Aasamaa et al., 2005; Nardini et al., 2010). Generally, *K*_leaf_ increases in developing leaves as the vasculature matures. In the weeks or months following its maximum, *K*_leaf_ begins to decline, by up to 80–90% at abscission (Aasamaa et al., 2005; Brodribb et al., 2005). Some authors have hypothesized that seasonal decline of *K*_leaf_ is a trigger for leaf senescence (Sack and Holbrook, 2006).

### IRRADIANCE

Variations in *K*_leaf_ due to changes in irradiance have now been reported in numerous plant species. In most cases, *K*_leaf_ is the lowest at low irradiance (<10 μmol photons m^-^^2^ s^-^^1^) or under darkness (Sack et al., 2002; Nardini and Salleo, 2005; Tyree et al., 2005). In sunflower (*Helianthus annuus*) for instance, *K*_leaf_ is reduced by 30–40% during the night compared to the day (Nardini and Salleo, 2005). Conversely, *K*_leaf_ can rapidly increase by several-fold in response to a high irradiance (up to >1000 μmol photons m^-^^2^ s^-^^1^; Sack et al., 2002; Lo Gullo et al., 2005). For instance, *K*_leaf_ was increased in the 30 min following a transition to high light in 6 out of 11 tropical plant species (Tyree et al., 2005). Light quality has also an important impact on leaf hydraulic properties. In silver birch (*Betula pendula*; Sellin et al., 2011) and cucumber (*Cucumis sativus*) leaves (Savvides et al., 2012), *K*_leaf_ was the highest under blue light, intermediate under white light, and the lowest under red light. It is of note that the *K*_leaf_ of *Arabidopsis* is also regulated by the light regime, but unlike the majority of species studied, it was increased by about 40% during the night and by twofold when night was extended by 5–15 h (Postaire et al., 2010).

More generally, *K*_leaf_ follows diurnal and seasonal rhythms. For sunflower and some tree species, *K*_leaf_ increased by up to two to threefold over a few hours from morning to midday and then declined by evening (Lo Gullo et al., 2005; Cochard et al., 2007). When sunflower plants were kept in the dark for several days, *K*_leaf_ continued to oscillate in phase with the subjective light period, indicating that these changes were driven by the circadian clock (Nardini and Salleo, 2005).

### DROUGHT STRESS

Leaves are able to sense and respond to various types of water shortage (Sack and Holbrook, 2006). When *Arabidopsis* plants were exposed to low air humidity (implying higher transpiration) a concomitant increase in *K*_leaf_ and whole plant hydraulic conductance was observed (Levin et al., 2007). ABA is also a central mediator of plant response to drought stress. Inhibiting effects of ABA on inner leaf water transport (*K*_leaf_) were recently revealed in *Arabidopsis* (Shatil-Cohen et al., 2011). In this study, ABA was fed to excised leaves through the xylem via transpiration. Pantin et al. (2013) confirmed these effects and showed that xylem-fed ABA decreased *K*_leaf_ and stomatal conductance (g_s_) in mutants that are known to be insensitive to ABA-induced stomatal closure. This suggested that the stomatal regulation was mediated via a hydraulic feedback in a tissue upstream of the stomata (Pantin et al., 2013).

### INTERACTION BETWEEN FACTORS ACTING ON LEAF HYDRAULIC CONDUCTANCE

Although most studies have addressed the effects of individual factors on *K*_leaf_, an integrated view of the dynamics and combined impacts of irradiance, leaf water status and development on *K*_leaf_ is now critically needed. This question was recently investigated in sunflower and three shrub species (Guyot et al., 2012). In each case, the amplitude of *K*_leaf_ response to light or leaf dehydration was positively correlated to the intensity of the other parameter. These properties may allow optimal adjustment of the leaf water status under contrasting conditions when light tends to enhance transpiration whereas soil water availability is declining. These few examples illustrate the diversity of physiological contexts leading to changes in *K*_leaf_. The following sections address the variety of molecular and cellular mechanisms involved and the physiological significance of these regulations.

## VASCULAR WATER TRANSPORT

### CONTRIBUTION OF THE VASCULAR PATHWAY TO LEAF HYDRAULIC CONDUCTANCE

**FIGURE 1:**
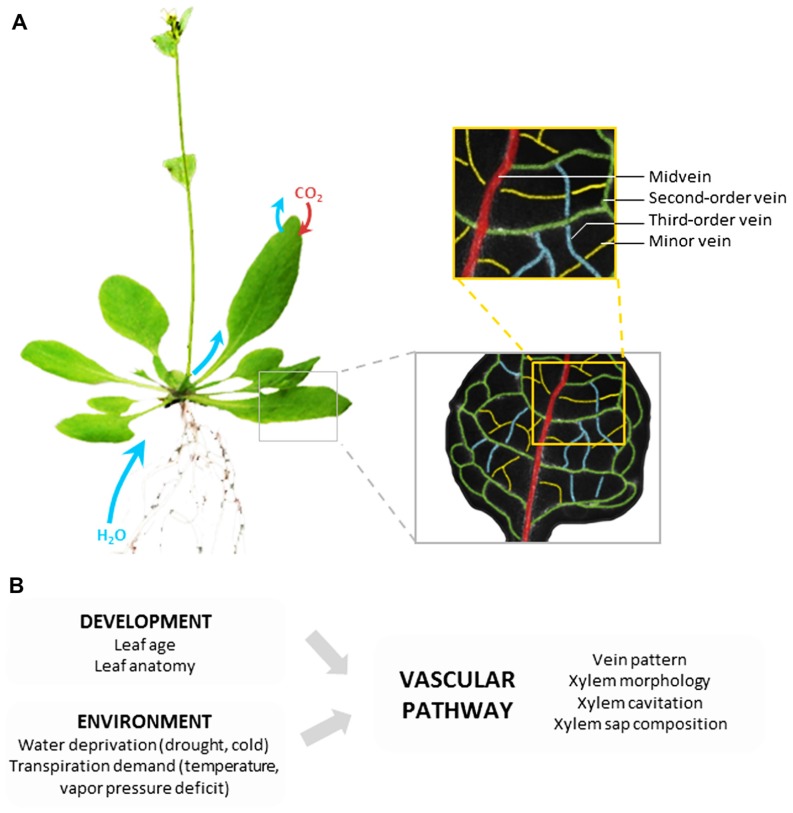
**Leaf vascular pathway and regulation of water transport**. **(A)** Based on the example of *Arabidopsis thaliana*, the figure shows that following uptake by roots and transfer to shoots, water is delivered throughout the whole leaf lamina by a highly organized network of veins. The non-living vessels form the leaf vascular pathway which is constructed according to a hierarchical order with midvein, second-order veins, third-order veins, and minor veins. The midvein runs from the petiole to the leaf apex, with second-order veins branching at regular intervals, and third-order veins branching on the latter. **(B)** The hydraulic resistance of the vascular pathway can be influenced by various developmental and environmental factors acting on the venation pattern or the indicated xylem properties.

The vascular pathway is composed of a highly structured network of differentiated (non-living) vessels that deliver xylem sap through the entire leaf, close to the evaporation sites. The minimization of transport distances out of the vascular pathway is one key feature of the hydraulic performance of leaves (Sack and Holbrook, 2006). In most dicots, venation is constructed according to a hierarchical order: midvein, second- and third-order veins, and finally minor veins that confer the reticulate pattern (**Figure 1A**). It is generally assumed that, whatever the leaf vascular anatomy, the bulk of transpired water follows the path of lesser resistance down the vein network, from midrib to minor veins before exiting the vessels (Sack and Holbrook, 2006). This means that water subsequently follows the vascular and extravascular paths.

The respective contributions of these two paths to *K*_leaf_ and to its variations have been the object of numerous studies. Models based on electrical analogies and using Poiseuille’s law have been developed to calculate the hydraulic conductance of leaf xylem networks (Lewis and Boose, 1995). They demonstrated the importance of hierarchy in the vein network to optimize water transport (Cochard et al., 2004; McKown et al., 2010). One first method to determine the contribution of veins to the leaf hydraulic resistance (*R*_leaf_, the inverse of *K*_leaf_) is to cut an increasing number of minor veins. The measured *R*_leaf_ progressively decreases and converges toward a stable value, the supposed vascular resistance (Sack et al., 2004; Nardini and Salleo, 2005). A second method consists in disrupting the living structures of the leaf by freezing or boiling the entire organ. The measured *R*_leaf_ is then reduced to its vascular component (Cochard et al., 2004) provided that the treatments do not alter the xylem vessel diameter or the extensibility of the walls. All these studies have revealed that the hydraulic resistances of the vascular and extravascular compartments are of the same order of magnitude. Either one may prevail, depending on species or environmental factors (Zwieniecki et al., 2002; Sack et al., 2004; Nardini and Salleo, 2005).

### VARIATIONS BETWEEN SPECIES

Leaf vascular anatomy is highly variable across species with respect to vein arrangement and density. The number, size, and geometry of the vascular bundles in the veins and of the xylem conduits within the bundles are also very diverse (Roth-Nebelsick et al., 2001). Yet, common principles of organization can be found, such as a global scaling between leaf size and vein characteristics. In particular, larger leaves have major veins of larger diameter, but lower length per leaf area, whereas minor vein traits are independent of leaf size (Sack et al., 2012; Sack and Scoffoni, 2013).

This great anatomic variability could explain to a large extent the dramatic differences in *K*_leaf_ observed between species. Several main trends relative to measured *K*_leaf_ variations have been validated through modeling (Cochard et al., 2007; McKown et al., 2010). Firstly, the conductance of the main veins appeared as a major limiting factor of *K*_leaf_. By contrast, the arrangement and density of these veins had a marginal impact on *K*_leaf_ (Sack and Frole, 2006) and would rather contribute to a uniform distribution of water across the lamina (Roth-Nebelsick et al., 2001; Zwieniecki et al., 2002) and avoid cavitation (Sack and Holbrook, 2006). Secondly, the *K*_leaf_ of plants with a higher minor vein density tended to be greater. This is not due to an increase in conductance of the xylem system *per se* (Cochard et al., 2004), but rather to an increase in the surface area for exchange of xylem sap with surrounding mesophyll and reduced distances in extravascular pathway (Roth-Nebelsick et al., 2001; Sack and Frole, 2006). A high vein density also favors water potential equilibration across the leaf and prevents the damage or blockage of higher-order veins (Sack and Scoffoni, 2013).

### THE CONSTRUCTION COST OF VASCULAR PATHWAYS

The development of a dense vein network represents a massive investment for the plant because lignified tissues are net carbon sinks that do not directly contribute to photosynthesis (Pantin et al., 2012). However, maximum net assimilation rate of photosynthesis depends on the capacity of the leaf vascular system to supply water to photosynthesizing mesophyll cells (Brodribb et al., 2007). Hydraulic modeling of leaves revealed that the conductivity and density profiles of veins of various orders contribute to optimizing the hydraulic efficiency of the xylem network. A high vein density only becomes economically viable compared to the photosynthetic costs when it is supported by a highly conductive low order venation. A high vein density limits the distance of photosynthate and water transport between veins, photosynthesizing mesophyll cells, and evaporative surfaces of the leaf (Amiard et al., 2005; Brodribb et al., 2007; McKown et al., 2010). Hence, the hydraulic properties of the leaf tissue play a fundamental role in linking leaf construction with photosynthetic capacity.

### ENVIRONMENTAL EFFECTS

It is of note that, beyond developmental factors, the functioning and hydraulic resistance of the vascular pathway depends on the plant growth conditions (Brodribb et al., 2010). The combined use of a xylem pressure probe and a Scholander–Hammel pressure bomb in intact maize (*Zea mays*) plants was used to demonstrate that leaf xylem pressure can change rapidly and reversibly with environmental modifications, such as light intensity or soil water potential (Wei et al., 1999). One striking consequence is water stress-induced xylem cavitations that result in marked reductions in *K*_leaf_ (Bucci et al., 2003; Nardini et al., 2003; Johnson et al., 2009). However, decrease of *K*_leaf_ in dehydrating pine needles (Cochard et al., 2004) appeared to be due to a collapse of tracheids. On the longer term, water shortage can interfere with leaf growth and xylem differentiation. In sunflower for instance, *K*_leaf_ was decreased in response to 20-day-long moderate or severe water stresses due to narrower xylem conduits (Nardini and Salleo, 2005). During winter, freeze–thaw cycles in vessels of woody plants can also result in xylem vessel embolism and/or wall collapse and therefore induce a significant decrease in *K*_leaf_ (Ameglio et al., 2001). Hence, different plant species may exhibit contrasting vulnerability to water stress- or winter-induced embolism, depending on the anatomy of their vessels.

The xylem sap composition, and in particular its potassium concentration, can interfere with the wall permeability of tracheids (Zwieniecki et al., 2001). These effects may be due to a shrinking and swelling of the pectin hydrogel forming the inter-vessel pit membranes. This mechanism which impacts *K*_leaf_ has been invoked to explain the effects of light on stem hydraulics in laurel and silver birch (*Betula pendula*; Nardini et al., 2010; Sellin et al., 2010).

In conclusion, the vascular compartment of leaves allows a broad range of hydraulic configurations between species, during development or in response to environment fluctuations (**Figure 1B**). As explained in the next sections, the extravascular structures can provide complementary means for rapid and reversible regulations of *K*_leaf_ (Sack and Holbrook, 2006).

## THE EXTRAVASCULAR COMPARTMENT

### WATER PATHWAYS INSIDE THE EXTRAVASCULAR COMPARTMENT

**FIGURE 2:**
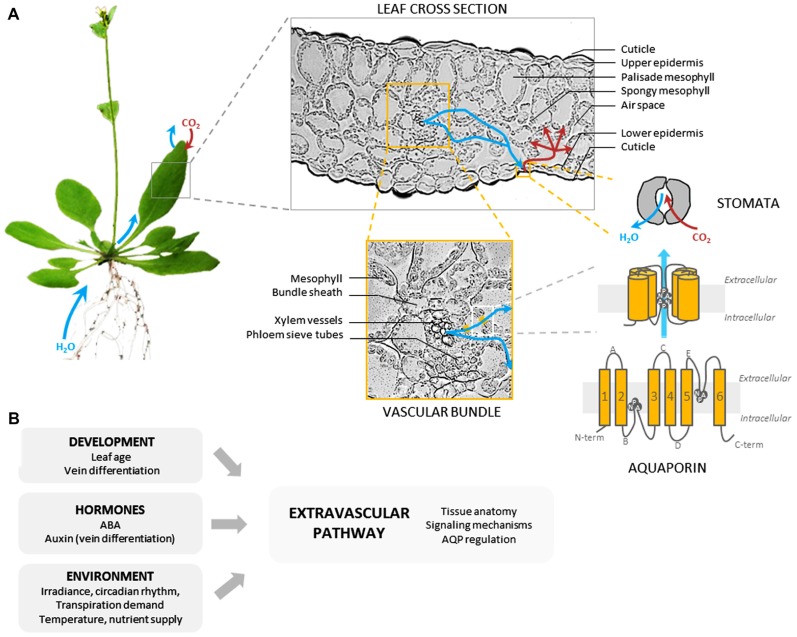
**Leaf extravascular pathway and regulation of water transport**. **(A)** The figure shows, using the *Arabidopsis* leaf as an example, the various components of the extravascular pathway, from whole organ to molecular levels. The pathway followed by water between xylem vessels and substomatal chambers is not entirely understood. Whereas the role of xylem parenchyma and bundle sheath is emerging (see text), the contribution of the mesophyll may depend on leaf anatomy. Water transport across living cells is mediated in part by water channel proteins called aquaporins (AQPs) formed by six α-helical transmembrane domains linked by five loops (A–E), and N- and C-terminal ends localized in the cytosol. Two specific, highly conserved structural motifs (NPA) are located in the pore and contribute to AQP selectivity. AQPs are expressed in all leaf living cells but preferentially in veins. **(B)** Various developmental and environmental factors act on the indicated components of the extravascular pathway to alter its hydraulic properties. AQP regulation occurs at various levels including gene expression, AQP trafficking and gating (see text).

The extravascular compartment includes all living tissues that transport water from xylem vessels to substomatal chambers. Following its exit from xylem conduits, water flows through xylem parenchyma cells and enters the bundle sheath made up of parenchymatous cells wrapped around the veins (Leegood, 2008). Water then crosses bundle sheath extensions or the mesophyll to reach the epidermis and evaporations sites, respectively. The location and surface area of the latter sites may vary according to leaf anatomy, some species having huge leaf internal airspaces (Brodribb and Feild, 2010; **Figure 2A**). Recently, a shift has been made from the simple idea that leaves can be reduced to a single pool of evaporating water to a more complex leaf representation with well-organized water pools separated by hydraulic resistances (Zwieniecki et al., 2007).

It is classically assumed that water can follow different paths to flow across living tissues, from cell-to-cell, through cell membranes (transcellular path) and plasmodesmata (symplastic path), or through the continuity of walls (apoplastic path; Steudle and Peterson, 1998). The relative contribution of these different paths in leaves is currently unclear and could vary according to species, leaf developmental stage (Voicu and Zwiazek, 2010), or physiological conditions (Sack et al., 2004; Nardini and Salleo, 2005; Cochard et al., 2007; Ye et al., 2008). Tissue anatomy can provide preliminary hints at these questions. Mesophyll tissues often have a low cell packing and are largely composed of airspaces. This, and experiments whereby apoplastic transport was traced using dyes such as 8-hydroxypyrene-1,3,6-trisulfonic acid (HPTS), have suggested that apoplastic water movement predominates during transpiration (Sack and Holbrook, 2006; Voicu et al., 2008, 2009). Water may cross cell membranes only for cell water homeostasis, during rehydration and expansion growth (Heinen et al., 2009). In contrast, the vascular bundles show physically tight cell layers (**Figure 2A**). In addition, recent work indicated that bundle sheath cells may have suberin lamellae and/or apoplastic barriers on radial walls, thereby decreasing the apoplastic flow of water (Lersten and Curtis, 1997). Thus, transcellular water flow may be critical at this site.

### THE DYNAMICS OF LEAF CELL WATER PERMEABILITY IN RESPONSE TO DEVELOPMENTAL AND ENVIRONMENTAL FACTORS

Several techniques have been developed to measure the water permeability of leaf cells and therefore dissect the functional behavior of the extravascular pathway. The cell pressure probe technique which gives access to cell water relation parameters in intact plant tissues has been applied to several cell types including the stomata, epidermis, mesophyll (Franks, 2003), and midrib parenchyma (Kim and Steudle, 2007, 2009). Since this technique is not applicable to small sized or deeply embedded cells, cell water permeability can also be characterized by means of osmotic swelling assays in protoplasts. The protoplasts are isolated according to their morphology or to cell-specific expression of fluorescent reporter proteins. This approach has been developed firstly in mesophyll protoplasts of various plant species (Ramahaleo et al., 1999; Morillon and Chrispeels, 2001; Martre et al., 2002) and more recently in protoplasts from *Arabidopsis* bundle sheath (Shatil-Cohen et al., 2011) and xylem parenchyma (Prado et al., 2013). In general, the water permeability of protoplasts is lower than in intact cells (Moshelion et al., 2004; Chaumont et al., 2005; Hachez et al., 2006, 2008; Volkov et al., 2007).

These techniques have first revealed that cell water permeability can vary according to leaf developmental stage (**Figure 2B**). In barley (*Hordeum vulgare*) and maize leaves, the water permeability of protoplasts isolated from the zones of emergence, elongation, and maturation was the highest in the former zone (Volkov et al., 2007; Hachez et al., 2008). A high cell water permeability may be beneficial during tissue expansion.

Measurements in individual leaf cells have also indicated that changes in *K*_leaf_ induced by environmental factors on the short-term may be mediated through changes in cell membrane water permeability (**Figure 2B**). For instance, the water permeability of individual parenchyma cells, as measured with a cell pressure probe in the midrib of maize leaves, was increased by up to threefold at low light intensities (Kim and Steudle, 2007). Other studies using protoplast swelling assays showed that, in maize, the leaf cell water permeability was the highest during the early hours of the day (Hachez et al., 2008). A similar approach revealed that diurnal leaf movements in rain tree (*Samanea saman*) and tobacco were linked to regulation of cell water transport in pulvini and petiole, respectively (Moshelion et al., 2002; Siefritz et al., 2004). The transpiration demand can also impact leaf cell water permeability. In *Arabidopsis* plants grown under various transpiring regimes or ABA treatments (Morillon and Chrispeels, 2001), an inverse relationship was found between mesophyll protoplast water permeability and the rate of plant transpiration which, however, could not be attributed to a direct action of ABA on the mesophyll. Bundle sheath cells seem to have, by contrast, a specific responsiveness to ABA which could explain the down-regulating effects of this hormone on *K*_leaf_ (Shatil-Cohen et al., 2011).

### HYDRAULIC LIMITATIONS IN THE EXTRAVASCULAR COMPARTMENT

The nature of the living cells that, within the leaf, oppose the major hydraulic resistance to the transpiration flow is still under debate (Cochard et al., 2004; Sack et al., 2004; Nardini and Salleo, 2005; Voicu et al., 2008). One recent approach made use of a non-invasive leaf pressure probe in *Arabidopsis* leaves (Ache et al., 2010). This new technique indicated that mesophyll cell turgor was markedly reduced at high transpiration rate, suggesting that an upstream structure, possibly the bundle sheath, was hydraulically limiting. In support for this, Shatil-Cohen et al. (2011) observed a correlation between the effects of ABA on *K*_leaf_ and the water permeability of protoplasts from the bundle sheath but not from mesophyll. This correlative approach was recently extended by Prado et al. (2013) who considered a larger set of vein protoplasts in *Arabidopsis* leaves. The data indicated that xylem parenchyma, in addition to bundle sheath, may be limiting during *K*_leaf_ regulation by light. A hydraulic limitation due to the xylem parenchyma was already suggested in maize leaf (Tang and Boyer, 2002). We note, however, that these conclusions may not apply to tobacco which showed no correlation between the hydraulic conductivities of whole leaves and bundle sheath cells (Lee et al., 2009). In addition, bundle sheath extensions which in some species link the bundle sheath to the epidermis and separate the leaf into chambers may influence the dynamics of *K*_leaf_ in response to irradiance and leaf water status (Sack and Scoffoni, 2013).

Altogether, water transport measurements in leaf cells have led to the realization that many of developmentally and environmentally induced variations of *K*_leaf_ may be explained through regulations of cell membrane water transport. Aquaporin (AQP) water channels are membrane proteins that facilitate the exchange of water across cell membranes and can be responsible for up to 95% of the water permeability of plant plasma membranes (Maurel et al., 2008). This explains the intensive research recently developed on the function and regulation of AQPs in leaves.

## AQUAPORINS IN LEAVES: TISSUE-SPECIFICITY AND PUTATIVE ROLES

### THE AQP FAMILY OF WATER CHANNEL PROTEINS

AQPs have a characteristically conserved structure with monomers (23–31 kDa) comprising six α-helical transmembrane domains linked by five loops (A–E) and N- and C-terminal ends localized in the cytosol (**Figure 2A**). AQPs assemble as tetramers, each monomer forming an individual transmembrane pore (Wang and Tajkhorshid, 2007). Plant AQPs show a great diversity, with >30 isoforms in higher plant species. They fall into at least four major homology subgroups that somehow reflect specific subcellular localizations (Maurel et al., 2008). For instance, the plasma membrane intrinsic proteins (PIPs) and the tonoplast intrinsic proteins (TIPs) represent the most abundant AQPs in the plasma membrane and in the tonoplast, respectively. The great diversity of plant AQPs also reflects a broad range of transport specificities (Tyerman et al., 2002). In addition to water, some AQP isoforms can transport non-polar solutes such as metalloids (Bienert et al., 2008), gases (Uehlein et al., 2003), or reactive oxygen species (ROS; Bienert et al., 2007; Dynowski et al., 2008), suggesting multiple functions, in water and nutrient transport, and cell signaling.

### TISSUE-SPECIFIC EXPRESSION OF AQPs AND PUTATIVE ROLES

**FIGURE 3:**
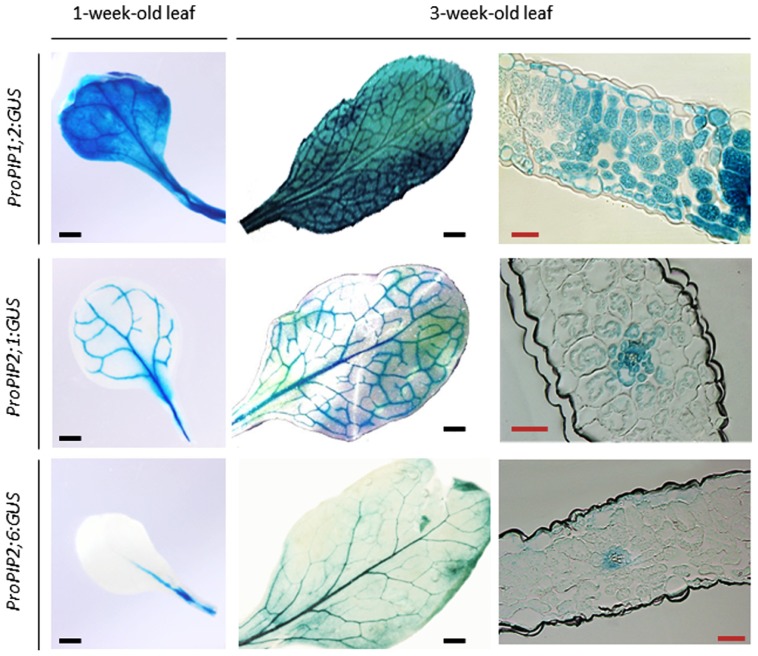
**Expression pattern of three highly expressed *PIP* genes during leaf development in *Arabidopsis***. The figure shows expression in transgenic plants of chimeric genes expressing a *β -glucuronidase* gene (*GUS*) under the control of *PIP* promoter sequences (*ProPIP*). The three indicated AQP isoforms (*At*PIP1;2, *At*PIP2;1, *At*PIP2;6) contribute to rosette hydraulic conductivity (Prado et al., 2013). Cross-sections show intense staining in the veins of each type of transgenic plants (black scale bar = 2.5 mm, red scale bar = 0.1 mm).

Expression profiling of the *AQP* gene family in several plant species has indicated that leaves are equipped with multiple AQP isoforms. By contrast to what was observed in pollen or seeds, no AQP transcript was strictly specific for leaves. In the *Arabidopsis* leaf, two *TIP* (*AtTIP1;2* and *AtTIP2;1*) and three *PIP* (*AtPIP1;2*, *AtPIP2;1*, and *AtPIP2;6*) genes are strongly expressed and *AtPIP2;6* shows preferential expression in this organ (Jang et al., 2004; **Figure 3**). Quantitative proteomics of plasma membranes purified from *Arabidopsis* leaves confirmed this pattern and showed that *At*PIP1;2, *At*PIP2;1, and *At*PIP2;7 were the most abundant among the nine PIPs isoforms detected (Monneuse et al., 2011).

Beyond these global studies, the marked cell-specific expression patterns of some isoforms can provide interesting hints at a variety of AQP functions in the leaf. In tobacco for instance, strong expression of a PIP1 homolog, *Nt*AQP1, was observed in spongy parenchyma cells of mesophyll, with the highest concentration around substomatal cavities (Otto and Kaldenhoff, 2000). AQPs may fulfill multiple roles in the mesophyll: transcellular water transport during transpiration, as suggested for *Nt*AQP1, but also cell osmotic adjustment under varying water demand, or CO_2_ transport (Otto and Kaldenhoff, 2000).

Yet, a preferential expression of AQPs in the vascular bundles was observed in many plant species, suggesting a special role for AQPs in delivering water from the vessels to the mesophyll (Kaldenhoff et al., 2008). In particular, bundle sheath cells were shown to have high PIP and TIP expression levels in rapeseed (*Brassica napus*; Frangne et al., 2001), *Arabidopsis* (Kaldenhoff et al., 1995; Prado et al., 2013), ice plant (*Mesembryanthemum crystallinum*; Kirch et al., 2000), Norway spruce (*Picea abies*; Oliviusson et al., 2001), maize (Hachez et al., 2008), and rice (*Oryza sativa*; Sakurai et al., 2008). This expression pattern is consistent with the observation that the bundle sheath is formed of highly compacted cells, with sometimes lignified or suberized cell walls (see Water Pathways Inside the Extravascular Compartment). Strong expression of AQPs in the xylem parenchyma has also been described in several species (Barrieu et al., 1998; Otto and Kaldenhoff, 2000; Sakr et al., 2003; Hachez et al., 2008; Prado et al., 2013). This site of expression may be crucial for radial cell-to-cell water movement during exit from the xylem vessels (Prado et al., 2013) and for osmotically driven water loading in xylem vessels during embolism refilling (Sakr et al., 2003; Secchi and Zwieniecki, 2010). AQPs were also found to be abundant in phloem companion cells (Kirch et al., 2000; Fraysse et al., 2005) suggesting a role in phloem sap loading and in maintaining vascular tissue functions under drought stress (Montalvo-Hernandez et al., 2008). Finally, AQPs are expressed in epidermis (Cui et al., 2008), trichomes, stomata (Heinen et al., 2009), and dividing cells (Barrieu et al., 1998) where their role still needs to be established.

This survey should not give a static view of AQP expression, which is constantly adjusted during leaf development. In maize and barley leaves for instance, some isoforms were highly expressed in young, elongating leaf tissues whereas others were preferentially expressed in fully developed, matured tissues (Wei et al., 2007; Hachez et al., 2008; Besse et al., 2011; Yue et al., 2012).

### INVOLVEMENT OF AQPs IN LEAF HYDRAULICS: PHARMACOLOGICAL AND GENETIC EVIDENCES

The contribution of AQPs to leaf water transport was first demonstrated using pharmacological inhibition. Treatment of mesophyll and bundle sheath protoplasts with mercury, which blocks AQPs through oxidation of Cys residues, resulted in a fivefold reduction in cell water permeability (Kaldenhoff et al., 1998; Shatil-Cohen et al., 2011). At the whole leaf level, mercury treatment decreased *K*_leaf_ by 33% in sunflower (Nardini and Salleo, 2005) and by around 40% in six temperate deciduous trees (Aasamaa et al., 2005). Although it is also rather unspecific and toxic, azide, which induces cell acidosis and a pH-dependent closure of PIPs (Tournaire-Roux et al., 2003), was used in *Arabidopsis* as an independent type of AQP blocker. The similar inhibiting effects of mercury and azide supported the idea that, in this species, PIPs truly contribute to the enhancement of rosette hydraulic conductivity under darkness (Postaire et al., 2010).

Given the lack of specific inhibitors, genetic approaches provide a more reliable approach for studying the physiological function of plant AQPs. *Arabidopsis* plants expressing *AtPIP1;2* or *AtPIP2;3* antisense transgenes, individually or in combination, showed in parallel to a reduced expression of PIP1s and/or PIP2s, a 5- to 30-fold reduction in water permeability of isolated mesophyll protoplasts (Kaldenhoff et al., 1998; Martre et al., 2002). The antisense lines also showed a leaf water potential and a *K*_leaf_ significantly lower than in control plants, only under water limiting conditions. The differences were stronger during re-watering, suggesting that AQP-mediated water transport was directly involved in leaf tissue rehydration (Martre et al., 2002). In tobacco, the phenotype of an antisense *NtAQP1* line suggested that this AQP is involved in the differential expansion growth of the upper and lower surfaces of the petiole during leaf unfolding (Siefritz et al., 2004). The contribution of individual AQPs to water leaf transport was thoroughly dissected in *Arabidopsis*. Plant lines carrying an individual T-DNA insertion in three out of four highly expressed *PIP* genes (*AtPIP1;2*, *AtPIP2;1, AtPIP2;6)* displayed, when grown in the dark, reduction in *K*_leaf_ by approximately 30%, similar to the reduction displayed by a corresponding triple *pip* mutants (Prado et al., 2013). Another study using a deuterium tracer method to assess water relocation in *Arabidopsis* showed that *K*_leaf_ was significantly reduced by about 20% in *pip2;1* and *pip2;2* knock-out plants (Da Ines et al., 2010).

## AQUAPORINS IN LEAVES: MODES OF REGULATION

### RESPONSE TO LIGHT AND CIRCADIAN RHYTHM

Understanding the molecular and cellular bases of AQP regulation in leaves, and therefore the modes of *K*_leaf_ regulation in response to developmental or environmental cues, represents an important focus in current research. Because of the dominating role of light and circadian rhythms in regulating *K*_leaf_, most of recent studies have been performed in this context. Combined HPM and quantitative RT-PCR analyses in detached walnut leaves revealed a positive correlation between the increase in *K*_leaf_ under high irradiance and the transcript abundance of two *PIPs*, *JrPIP2;1* and *JrPIP2;2* (Cochard et al., 2007). The light-dependent stimulation of *K*_leaf_ in European beech (*Fagus sylvatica*) and pedunculate oak (*Quercus robur*) was also associated to enhanced expression of *PIP1* genes (Baaziz et al., 2012). Diurnal oscillations in expression of *NtAQP1* in tobacco leaf petioles (Siefritz et al., 2004), *SsAQP2* in motor cells of *Samanea saman* leaves (Moshelion et al., 2002), and most of *ZmPIP* genes in maize leaves (Hachez et al., 2008) were correlated to changes in water permeability of corresponding protoplasts. However, light-dependent *K*_leaf_ was not associated to any AQP transcriptional control in certain species such as bur oak (Voicu et al., 2009). Quantitative proteomic analysis in the *Arabidopsis* rosette showed that the abundance of each of the nine detected PIP isoforms was perfectly stable regardless the light regime (Prado et al., 2013). In contrast, the diphosphorylation of *At*PIP2;1 at two C-terminal sites (Ser280 and Ser283) was enhanced by twofold under the same conditions. Whereas the rosette hydraulic conductivity of a *pip2;1* knock-out mutant had lost any responsiveness to the light regime, expression in the same background of phosphomimetic and phosphorylation deficient forms of *At*PIP2;1 demonstrated that phosphorylation at Ser280 and Ser283 was necessary for *K*_leaf_ enhancement under darkness (Prado et al., 2013).

### WATER STRESS

Plants can undergo water stress in response to numerous environmental constraints such as drought, low atmospheric humidity, salinity, or cold. Studies trying to relate physiological responses to water stress with expression profile of AQPs have led to contrasting results depending on the time course and intensity of water stress (Tyerman et al., 2002; Galmés et al., 2007). Some studies have shown, however, that water stress can coordinately alter AQP expression and activity in the leaf. In grapevine (*Vitis vinifera*) under reduced irrigation for instance, the *K*_leaf_ was decreased by about 30% together with the expression of *VvTIP2;1* and *VvPIP2;1* (Pou et al., 2013). A low humidity treatment also induced a coordinated up-regulation of many *PIP* and *TIP* genes in rice leaves (Kuwagata et al., 2012). Enhanced expression of some AQPs may also support a role in embolism refilling. For instance, *JrPIP2* which was highly expressed in vessel-associated cells of walnut leaves during the winter period (Sakr et al., 2003).

Proteomic approaches have provided complimentary insights into the mode of AQP regulation under drought. A label-free quantitative shotgun approach in rice leaves under moderate or extreme drought or re-watering conditions showed that most of the nine AQPs identified were responsive to drought, with six decreasing rapidly during plant re-watering (Mirzaei et al., 2012). Phosphoproteomic analyses of *Arabidopsis* seedlings indicated that the C-terminal phosphorylation of *At*PIP2;1 decreased after 30 min of an ABA treatment (Kline et al., 2010). This observation is consistent with the down-regulating effects of ABA on *Arabidopsis K*_leaf_ through a mechanism that involves bundle sheath cells (Shatil-Cohen et al., 2011; Pantin et al., 2013). Thus, similar to what was described in leaves under changing light (Prado et al., 2013), altered phosphorylation of AQPs in veins may act on their trafficking and gating (Törnroth-Horsefield et al., 2006; Prak et al., 2008; Eto et al., 2010) to adjust leaf hydraulics during plant response to drought. The decreased phosphorylation of spinach *So*PIP2;1 following a hyperosmotic treatment in leaf fragments (Johansson et al., 1996) was initially interpreted in the context of leaf cell turgor regulation, whereby an enhanced activity (phosphorylation) of *So*PIP2;1 would favor water influx under fully hydrated conditions. It could also correspond to a water stress-dependent regulation of *K*_leaf_.

### SIGNALING MECHANISMS ACTING UPSTREAM OF AQP REGULATION

The signaling mechanisms that act upstream of leaf AQP regulation now represent a critical challenge for future research. They likely involve ROS and calcium (Ca^2^^+^), which both display specific signatures during leaf response to environmental or hormonal stimuli.

Hydrogen peroxide (H_2_O_2_) is now recognized as a potent regulator of plant AQPs. H_2_O_2_ perfusion via the petiole decreased by up to 30-fold the water permeability of epidermal and parenchyma cells, in wandering jew (*Tradescantia fluminensis*; Ye et al., 2008) and maize (Kim and Steudle, 2009) leaves, respectively. A ROS-dependent down-regulation of AQPs has also been invoked to explain the inhibition at high light intensities of the hydraulic conductivity of parenchyma cells, in the midrib tissues of maize leaves (Kim and Steudle, 2009). The mode of action of ROS on water transport is still debated. Hydroxyl radicals produced from exogenously supplied H_2_O_2_ may act on AQP gating by direct oxidation (Henzler et al., 2004). Such effects were not observed in *Arabidopsis* whereby H_2_O_2_ triggers a cell signaling cascade ultimately leading to PIP down-regulation, through altered phosphorylation and/or cellular internalization (Boursiac et al., 2008; Prak et al., 2008).

Ca^2^^+^plays key structural and signaling roles in plants. It can directly inhibit PIP activity *in vitro* (Gerbeau et al., 2002; Alleva et al., 2006; Verdoucq et al., 2008) by a molecular mechanism that involves Ca^2^^+^ binding to the cytosolic side of the AQP to stabilize its closed conformation (Hedfalk et al., 2006; Törnroth-Horsefield et al., 2006). This effect has not yet been related to any physiological process in the plant. Plant AQPs can also undergo Ca^2^^+^-dependent phosphorylation, which in turn increases their water channel activity. For instance, *in vitro* phosphorylation of spinach leaf PM28A (*So*PIP2;1) was mediated by a plasma membrane-associated protein kinase that was strictly dependent on submicromolar concentrations of Ca^2^^+^ (Johansson et al., 1996; Sjövall-Larsen et al., 2006). This and other protein kinases acting on leaf AQPs still await biochemical and molecular characterization. An integrative model that links the water flow pathways and Ca^2+^ distribution in leaves was recently proposed (Gilliham et al., 2011). According to this model, the delivery of apoplastic Ca^2^^+^ and its storage could determine most of hydraulic regulations involving leaf AQPs.

## INTEGRATION AND MANIPULATION OF LEAF HYDRAULICS

### LEAF HYDRAULIC CONDUCTANCE AND WATER STATUS

Because the leaf water status is at the cross-road of fundamental physiological functions including carbon fixation and growth, its manipulation or genetic improvement could help optimize the entire plant performance, including yield and adaptation to environmental constraints, over short and long time scales. However, several important principles first need to be emphasized to understand the integrative aspects of plant leaf hydraulics and the potential and possible pitfalls of its manipulation.

The present review addressed plant leaf hydraulics, essentially by looking at the multiple facets of *K*_leaf_. It is of note that, in plants under transpiring conditions, the dominating resistance for water transport across the plant does not operate in inner leaf tissues but on vapor diffusion, through stomata and at the leaf surface. Thus, the direct impact of *K*_leaf_ on the intensity of the leaf transpiration may be marginal. The physiological importance of *K*_leaf_ should not be underestimated, however, since under a fixed transpiration regime, *K*_leaf_ strongly impacts on the hydration status of the inner leaf tissues (Tsuda and Tyree, 2000). As explained below, leaf hydraulics has a great significance for growth, due to crucial links between this process and leaf water potential. Water potential maintenance in inner leaf tissues is also linked to hydraulic conductance of vessels and stomata and, as a result, interferes with the transpiration flow. For instance, stimuli such as light that enhance *K*_leaf_ actually promote water supply to the inner leaf tissues to prevent an excessive drop in water potential throughout the transpiring leaf (Tsuda and Tyree, 2000). This may help reduce tensions and avoid cavitations in xylem vessels. Conversely, a hydraulic limitation in veins, which can typically be enhanced by ABA-dependent down-regulation of AQPs in these territories, can result in a hydraulic signal to promote stomatal closure in plants under water stress (Pantin et al., 2013). This example emphasizes the fundamental interplay that exists between leaf water potential, *K*_leaf_ and g_s_.

### AQPs AND HYDRAULIC CONTROL OF LEAF GROWTH

While most of the water absorbed by the plant is lost by transpiration, a minor fraction is retained for supporting leaf growth (Pantin et al., 2012). Leaf expansion growth primarily results from a fine interplay between cell wall relaxation and cell water potential, which both determine the rate of water inflow (Cosgrove, 1987). It is therefore highly sensitive to the leaf water status and has to be protected from environmental disturbances.

The finding of growth-induced water potential gradients (Fricke, 2002; Tang and Boyer, 2002) provided the first direct evidence that leaf growth can be hydraulically limited. This idea is also supported by enhanced function of AQPs in expanding tissues. In cereal leaves for instance, cell water permeability was higher in the elongation zone than in the emerged non-growing zone (Volkov et al., 2007; Hachez et al., 2008). Preferential expression of AQP isoforms in leaf expanding tissues was described in several plant species (Wei et al., 2007; Hachez et al., 2008). This pattern was not restricted to plasma membrane AQPs since expression of *AtTIP1;1* was associated with cell enlargement in *Arabidopsis* leaves (Ludevid et al., 1992) and enhanced by the growth-promoting hormone gibberellic acid (GA3; Phillips and Huttly, 1994). Vacuolar AQPs may favor the differentiation of a large central vacuole that is characteristic of fully elongated cells (Ludevid et al., 1992). Whole plant measurements have also provided evidence for hydraulic limitation of leaf growth. In *Arabidopsis*, it occurs during leaf ontogeny, with leaf growth becoming slower during the day than at night (for a review, see Pantin et al., 2012). In maize, leaf growth was highly sensitive to alterations of inner plant hydraulic conductance, through pharmacological inhibition of AQPs (Ehlert et al., 2009) or genetic alteration of ABA biosynthesis which in turn altered AQP expression (Parent et al., 2009).

In summary, a hydraulic resistance between vascular and peripheral expanding tissues may result in marked growth-induced water potential gradients, which would in turn collapse cell turgor and result in an immediate growth arrest. Thus, high AQP-mediated cell water permeability can be highly beneficial to enhance cell-to-cell water transport in expanding tissues. Under water stress conditions, however, solute deposition rate in the elongation zone may become the limiting factor to sustain water inflow, turgor and ultimately growth (Fricke and Peters, 2002). There are now numerous reports showing that AQP deregulation can lead to enhancement of plant growth, but the reasons behind must be more complex than a direct alleviation of hydraulic limitations for growth. For instance, overexpression of *At*PIP1;2 in tobacco plants led to a significant increase in plant growth rate, leaf transpiration rate, stomatal density, and photosynthetic efficiency under favorable growing conditions (Aharon et al., 2003). By contrast, these plants showed a very poor response to water deprivation with enhanced leaf wilting, indicating that stomatal deregulation was the primary cause of altered growth in transgenic materials. Some transgenic strategies were more successful to optimize growth in drought conditions. For instance, overexpression of *OsPIP1;3* under the control of a stress-inducible promoter in a drought-sensitive cultivar of rice, resulted in a higher leaf water potential and transpiration rate in water stress conditions (Lian et al., 2004). This indicates that this AQP can indeed play a role in drought resistance and ultimately promote plant growth.

This kind of observations has now found a better interpretation frame by considering the anisohydric vs. isohydric water management strategies (Sade et al., 2012). Isohydric plants exert a strict stomatal control to maintain midday leaf water potential, independent of environmental constraints. Anisohydric plants have a more risky strategy and keep their stomata open under conditions of water shortage, to maintain photosynthetic assimilation and growth, but at the expense of leaf water potential maintenance. This strategy, which requires improved tissue hydraulic performance was associated to enhanced expression of certain tonoplast AQP isoforms in leaves. Overexpression of a TIP homolog in tomato (Sade et al., 2010) increased mesophyll protoplast water permeability and transpiration, especially under water limiting conditions. In addition, a strong relation between *TIP2;1* expression, *K*_leaf_ and g_s_ was observed in grapevine under various irrigation regimes (Pou et al., 2013).

### AQPs, CARBON FIXATION AND GROWTH

Following the initial phase of turgor-driven cell expansion, a proper supply of carbon and therefore efficient photosynthesis are necessary for new cell wall deposition and an overall increase in dry matter (Pantin et al., 2012). Thus, the ability of some plant AQPs to transport CO_2_, in addition to water, may also be highly relevant to their beneficial role in plant growth. In particular, functional expression in oocytes or yeast of a tobacco PIP AQP, *Nt*AQP1, has shown that this AQP can enhance membrane permeability to gaseous CO_2_ (Uehlein et al., 2003). Immunological and translational fusion approaches further showed that *Nt*AQP1 was present in guard cells and mesophyll cells, where it localized to both the plasma membrane and in the inner chloroplast membranes. The latter localization is particularly suggestive of a role in CO_2_ assimilation (Uehlein and Kaldenhoff, 2008).

In transgenic tobacco plants with altered expression of *NtAQP1*, the rate of ^14^C incorporation in leaf disks fed with ^14^CO_2_ (Uehlein et al., 2003), the intensity of gas exchange, chlorophyll fluorescence, and ^13^C discrimination (Flexas et al., 2006) were positively correlated to the level of *NtAQP1* expression. These results were interpreted to mean that *Nt*AQP1 functions as a CO_2_ channel in the mesophyll. These initial observations have now been extended to rice (Hanba et al., 2004) and *Arabidopsis* (Heckwolf et al., 2011). In the latter study, *Arabidopsis pip1;2* knock-out plants displayed a reduction by 40% of their mesophyll conductance (*g*_m_) to CO_2_. With respect to previous reports, this work defines a clear molecular and genetic context in which to address the function of PIPs in CO_2_ transport. In view of other possible contributors of *g*_m_ such as cell walls and carbonic anhydrases (Evans et al., 2009), it remains to be understood, however, how a single AQP isoform can contribute up to 40% of *g*_m_. Also, it is intriguing that the *At*PIP1;2 isoform was also identified as an important component of root and leaf hydraulics (Postaire et al., 2010). Thus, much remains to be learnt about the interplay and regulation of water and CO_2_ transport by AQPs. The possible coupling of tissue hydraulics with growth and carbon assimilation provides unique research perspectives in plant integrative biology.

## CONCLUSION

Recent research indicates that the veins, and the AQPs that are expressed in these territories, represent key determinants of leaf hydraulics. Understanding how the vascular architecture of leaves optimizes their hydraulic behavior or, in other words, understanding the adaptive value of leaf venation according to species and/or natural habitats represents an important challenge for future studies. Besides studies on xylem differentiation, a better knowledge of the function and regulation of the numerous AQP homologs expressed in plant leaves is also critically needed to understand how multiple environmental factors such as day/night cycles or water stress act alone or in combination to alter leaf hydraulics. While a role for AQPs in phloem loading, leaf movement and CO_2_ transport is emerging, we also anticipate that genetically altered plants will help decipher these and other new AQP functions. Finally, integrative studies have shown how the hydraulics of inner leaf tissues can have a strong impact on the dynamic responses of leaf water potential and stomata, and as a consequence on plant carbon economy and leaf expansion growth. These studies point to the power but also complexity of biotechnological strategies where plant AQP function is manipulated to potentially improve plant growth and tolerance to water stress.

## Conflict of Interest Statement

The authors declare that the research was conducted in the absence of any commercial or financial relationships that could be construed as a potential conflict of interest.
